# Identifying novel biomarkers through data mining—A realistic scenario?

**DOI:** 10.1002/prca.201400107

**Published:** 2015-01-12

**Authors:** Johannes Griss, Yasset Perez‐Riverol, Henning Hermjakob, Juan Antonio Vizcaíno

**Affiliations:** ^1^ European Molecular Biology LaboratoryEuropean Bioinformatics Institute (EMBL‐EBI)Wellcome Trust Genome CampusHinxtonCambridgeUK; ^2^ Division of ImmunologyAllergy and Infectious DiseasesDepartment of DermatologyMedical University of ViennaAustria

**Keywords:** Bioinformatics, Biomarker, Databases, Data mining, Mass spectrometry

## Abstract

In this article we discuss the requirements to use data mining of published proteomics datasets to assist proteomics‐based biomarker discovery, the use of external data integration to solve the issue of inadequate small sample sizes and finally, we try to estimate the probability that new biomarkers will be identified through data mining alone.

**Abbreviations**
GPDE: Griss Proteomics Database Engine
GPMDB: Global Proteome Machine Database
MIAPE: Minimum Information About a Proteomics Experiment
NIST: National Institute of Standards and Technology
PASSEL: PeptideAtlas SRM Experiment Library
PSA: prostate‐specific antigen
PSI: Proteomics Standards Initiative
PX: ProteomeXchange

Targeted personalized treatment options have become a major hope of clinical (e.g. cancer) research within the past years 1. Success stories such as herceptin 2 in breast cancer and BRAF^V600E^ inhibitors 3 in melanoma have kindled the quest to identify novel biomarkers for diagnosis, patient stratification, and personalized treatment options. Nevertheless, it is well known that biomarkers must be identified and used with care. This is best exemplified by the controversy around the usage of the prostate‐specific antigen (PSA) to detect prostate cancer 4. In fact, after several years, it was found that patient survival did not improve after PSA‐based prostatectomy 5.

The clinical validity of any biomarker is based on its sensitivity (the ability to identify “sick” people) and specificity (the ability to differentiate between “healthy” and “sick” people), next to the disease's or phenotype's prevalence. PSA, for example, may lead to up to 80% false‐positive test results, which caused unnecessary treatments 6. Estimating a biomarker's specificity is considerably more complex than to assess its sensitivity but equally important for its clinical success. Correctly assessing the specificity is directly dependent on the used control samples 7. To correctly estimate the specificity, it is not sufficient to only use “healthy” control samples but also samples from closely related diseases 7. Analyzing such a large enough number of heterogeneous control samples is at present outside the scope of most studies.

Genomics is frequently perceived as the “older brother” of proteomics 8. Only recently, Yuan et al. published a study identifying novel somatic mutations in clinical relevant genes 9, without analyzing a single new sample. Instead, for their study they reanalyzed multiple studies from The Cancer Genome Atlas (http://cancergenome.nih.gov) focusing on 12 different tumor types. Through the considerably increased number of analyzed samples, the authors were able to detect less frequent mutations that could not be detected in the smaller, original studies. A second, commercial example is GENEVESTIGATOR® by Nebion (http://www.nebion.com): It is based on a large database of reprocessed and manually curated public genomics datasets. Through this reanalysis, Nebion claims that it is possible to directly compare the gene expression profiles of the individual samples. Based on these, researchers can identify published samples with similar gene profiles as their own, as well as do an in silico analysis of genes of interest.

These two projects inherently raise the question if, and if possible when, such approaches using directly published data at the sample level will be feasible for proteomics experiments. Potentially, may it even be possible to conduct proteomics studies that only use existing data as in silico control samples? In the discussion of their manuscript, Yuan et al. highlight two important issues they encountered that equally apply to proteomics data in the public domain: (i) the lack of sufficient metadata to allow a more focused reanalysis of available data; and (ii) the more general problem that many studies focus on *p‐*values rather than on the study's magnitude. This first problem, the lack of metadata, has been highlighted many times before as an issue for proteomics data as well 7. Especially in clinical research, it is imperative that the analyzed samples are well characterized. It is not sufficient to know, for example, that a patient had a certain tumor. It is equally important to know, for example, the tumor stage, the tumor's known molecular characteristics, as well as any possible pretreatments. The second problem addressed by the authors, the focus on *p*‐values rather than on sample size, has led to an increasing number of studies that report the identification of novel biomarkers, which are then disproved in subsequent expensive clinical studies 7. In practice, authors often argue about a potential biomarker's clinical use based on its highly significant *p*‐value disregarding an inadequate number of analyzed samples. Additionally, we often see studies that analyzed only a handful of samples while measuring hundreds of analytes. This inevitably leads to the danger of an overfitting of biomarker associations 10.

Correspondence concerning this and other Viewpoint articles can be accessed on the journals' home page at: http://viewpoint.clinical.proteomics‐journal.com

Correspondence for posting on these pages is welcome and can also be submitted at this site.

In this article, we discuss the current availability of proteomics data in the public domain, the requirements to use data mining of these published proteomics datasets to assist proteomics‐based biomarker discovery, the use of external data integration to solve the issue of inadequate small sample sizes, and finally, we try to estimate the probability that new biomarkers will be identified through data mining alone.

Historically, public data deposition in proteomics has been much less common than in other biological fields. In fact, public availability of proteomics data has only recently taken a big step forward with the foundation of the ProteomeXchange (PX) consortium 11. There, the most prominent proteomics repositories including the PRIDE database 12 and PeptideAtlas 13 joined forces to standardize the submission and dissemination of MS‐based proteomics data. Submissions to PX are publicly announced to all interested parties and contain raw data as well as processed results (primarily identification data, in some cases also quantification). In the current implementation of the data workflow, PRIDE is the initial point of submission for MS/MS experiments, while PeptideAtlas provides a repository for SRM datasets called PASSEL (PeptideAtlas SRM Experiment Library) 14. Another repository named MassIVE (http://proteomics.ucsd.edu/service/massive/) has very recently joined PX as well (Fig. 1). The collaboration of these major proteomics resources has resulted in a rapid increase of publicly available proteomics datasets. Other proteomics repositories will not be discussed here in such high detail (reviewed in, e.g., 15, 16, 17).

**Figure 1:**
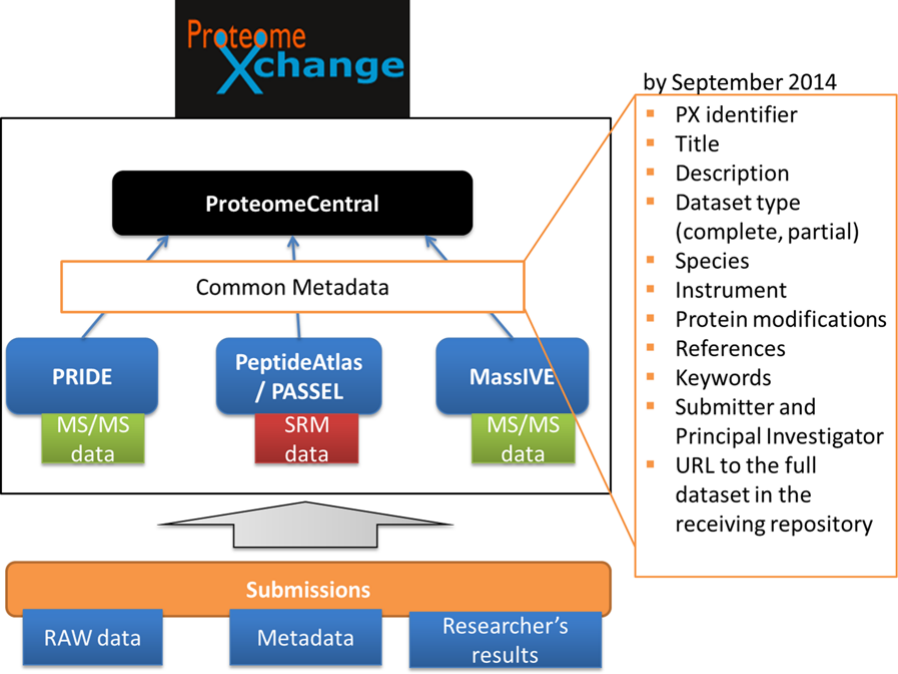
Current structure of the ProteomeXchange data workflow. Submissions have to contain the experiment's raw data, metadata, and the researcher's processed results (e.g. the identification data). ProteomeCentral is the portal for all ProteomeXchange datasets, independently from the receiving repository.

At the time of writing (September 6, 2014), PX stores 147 public clinical datasets (111 in PRIDE, 35 in PeptideAtlas/PASSEL, and 1 in MassIVE, see Supporting Information Table 1). This list was compiled based on the information available (e.g. title of the dataset, abstract) in the PX central portal (called ProteomeCentral, http://proteomecentral.proteomexchange.org/, Fig. 1). It is important to highlight that the metadata available in ProteomeCentral are, in most cases, only a subset of the metadata stored in the original repository (the minimum common denominator). However, these additional metadata are not available through ProteomeCentral, although the record in the original repository (PRIDE, PeptideAtlas/PASSEL, or MassIVE) is linked from there. Nevertheless, when writing this manuscript, we tried to simulate the process an average user (nonexpert in proteomics resources) would take at present to compile the list of datasets based on this information. The list presented here was manually curated and further classified in clinical subcategories (Fig. 2). As a result, the three most prominent dataset types found in human samples were datasets focused on the characterization of cancer, various tissue types (other than cancer), and on the study of other diseases (Fig. 2). The way this list had to be generated highlights the consequence of missing structured metadata. To compile the list, it was necessary to read through the description text of all human PX submissions as only the analyzed sample's species was always available in ProteomeCentral.

**Figure 2:**
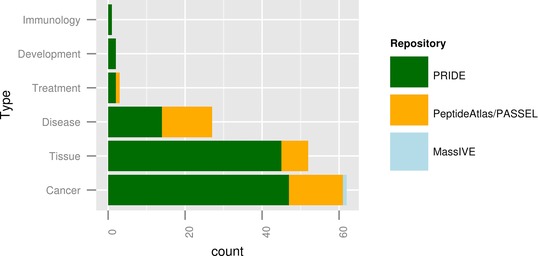
Frequency of clinical dataset types found in Proteome‐Xchange per receiving repository (by September 6, 2014).

To overcome the problem of insufficient metadata, the PSI (Proteomics Standards Initiative) developed the Minimum Information About a Proteomics Experiment (MIAPE) guidelines 18. These guidelines define the necessary minimum metadata to allow the retracing of all analytical steps. Additionally, the PSI has developed several vendor‐neutral standard data formats 19, 20, 21, 22 to overcome the representation heterogeneity of proteomics data. These standard file formats all support the reporting of MIAPE compliant metadata and can additionally hold structured metadata about the analyzed sample. In practice, almost no dataset stored in public repositories is annotated to the level specified by the MIAPE guidelines. But even if this were the case, this would not ensure by any means that metadata about the analyzed samples are complete. This is due to the fact that, generally, the MIAPE guidelines do not require extensive annotation about the actual samples.

Of the detected clinical PX datasets, 71 (48%) included the processed results in a standardized data format (the so‐called PX “complete” submissions) 11. To access more detailed metadata of the “complete” submissions, the authors had to access the linked dataset in PRIDE, visit the PRIDE web, download the file using the PRIDE Inspector tool 23, or directly inspect a generated summary file in mzTab format, available in the PRIDE FTP server. mzTab was recently developed by the PSI to explicitly facilitate proteomics data reuse by a wider audience including clinicians 22. It is a simpler tab‐delimited file format representing the *final* results of a proteomics experiment. Since mzTab files are tab‐delimited text files, they can be viewed and to a certain extent edited using standard software such as Microsoft Excel®. Looking at the large growth rate of PX, such an analysis will soon become prohibitively time consuming and even impossible for users that do not have certain computer skills to automate metadata retrieval. The other type of PX submissions, so‐called “partial” submissions (containing the processed results in a nonstandard format), include at present only basic metadata in a structured form, such as the sample's species, the used mass spectrometer, and software. In any case, the most critical pieces of information for any biological or clinical data reuse, the experimental protocol and information about the analyzed samples, were generally missing, incomplete, or available in a nonstructured free‐text format.

Even though a significant number (around 50% of the public clinical datasets at the time of writing) of submissions are using standard data formats (PX “complete” submissions), we are still at great risk to continue to lack vital metadata. A major reason for this is that the software generating proteomics results is mostly not aware of the metadata associated with the analyzed sample. Thereby, even if a standard file format is supported, the initially generated files do not contain any metadata about the sample. In many cases, especially in clinical research, this information is not available to the laboratory or core facility performing the proteomics experiment as the study is conducted by a clinician. This can be seen in the fact that the available annotated files in PX often contain detailed, manually annotated information about the mass spectrometer and its settings but generally very little information about the analyzed sample. Therefore, we desperately need methods that enable data submitters to easily annotate their processed result files. As an important step to alleviate this problem, work on such a tool for mzTab is planned by the PRIDE team and will hopefully help to increase the amount of metadata available in submitted files.

Nevertheless, in our experience there is always a balance between the required amount of metadata and the willingness of researchers to submit their data. This balance was taken into account when creating the initial PX data workflow. The focus was put on making it as practical and easy as possible for researchers to make their data publicly available and accessible. In our opinion, this was needed since the primary objective was to change the “culture” of data sharing in the field and public data deposition was still scarce. In this context, annotating processed result files is additional work for the submitter—work that, in most cases, is not perceived to be of direct benefit to them. Therefore, the types of metadata enforced through repository requirements must be defined with great care. As mentioned before, the current MIAPE guidelines primarily focus on the reproducibility of the MS experiments. This aspect is important for the retracing and reviewing of experiments but neglects the aspect of data reuse. With the continuous maturation of proteomics protocols, the increasing use of PX, and the increase of submitted data, we must justify the growing resources required to keep these data available. Therefore, we must shift our focus from data review to data reuse.

In addition to MS/MS data, PX also fully supports the submission of targeted SRM experiments through PeptideAtlas/PASSEL as the initial point of submission. Targeted experiments can be used to identify and quantify the predefined proteins of interest. Therefore, the possibilities to reuse the data differ distinctly from untargeted MS/MS experiments. The core benefit of such data is the availability of transitions necessary to plan new SRM experiments. Multiple resources, for example SRMAtlas (http://srmatlas.org/), already use public data to provide transition lists for a large number of proteins from multiple organisms. The direct comparison of SRM data is only sensible if a comparable set of proteins was analyzed. Therefore, in our opinion, the reuse of this valuable data faces fewer challenges as compared to untargeted MS/MS data but inherently cannot lead to new identifications in the published datasets. Additionally, differences in the used data analysis do not impede the reuse of gathered results. Therefore, we believe that the reuse of targeted proteomics data is, as seen through SRMAtlas, already successfully happening on a daily basis. Thus, we focus this viewpoint on untargeted approaches as these have greater, unsolved challenges for data reuse, which can potentially lead to new identifications in already analyzed datasets.

In this context, data mining of proteomics results can be performed at two levels: as a complete reanalysis of the mass spectrometer's raw files, or as the integration of known identification details (peptides and/or proteins). Several resources exist that generate combined datasets based on the reanalysis of proteomics experiments with the most prominent being PeptideAtlas 13 and GPMDB (Global Proteome Machine Database) 24. Both provide access to multiple datasets that were reprocessed with their own respective pipeline. Additionally, both resources employ dedicated algorithms to directly control the protein false discovery rate. This is essential as the combination of datasets from heterogeneous origins otherwise can lead to a vast increase of incorrectly identified proteins 25, 26, 27. However, the same problem mentioned before applies since only a part of the metadata from the analyzed datasets is available in an organized, machine‐readable structure. Therefore, there is no simple method to quickly assess whether the potential biomarkers found in a given study were already identified in other conditions. More importantly, it is not possible to quickly identify samples that could be incorporated as, for example, controls in one's own study.

In addition, a recent example of a complete reanalysis of multiple proteomics datasets is the “draft of the human proteome” by Wilhelm et al. 28. The authors enriched their own experiments with publicly available datasets stored in proteomics resources such as PX (corresponding to around 40% of their data). For these datasets, the authors reanalyzed the raw files. The results (about 1.1 billion peptide spectrum matches) were made available in a new database called “ProteomicsDB.” ProteomicsDB is run on a computational resource with 2 terabytes (TB) of random access memory (RAM) and 160 central processing units (CPUs). The computational effort required to analyze the actual experiments is not mentioned. These numbers clearly highlight the computational resources required to perform such meta‐analysis, which are prohibitive for most research groups. Wilhelm et al. followed a less‐stringent approach for calculating the protein FDR when combining their datasets. The danger is to obtain larger numbers of likely false‐positive protein identifications, as it has been shown for the olfactory receptors in ProteomicsDB 27. This highlights the issues related to the heterogeneity in data reprocessing pipelines.

An alternative method to integrate datasets is to directly rely on the originally reported identifications. In theory, through the PSI's standardized data formats, this task should be easy. Unfortunately, as mentioned before, only a subset of the datasets available in PX reports identification data in one of these formats. An additional obstacle is the fact that protein sequence databases are constantly updated and thereby changing. This leads to protein accessions, the primary identifier of proteins, being changed, merged, demerged, or even deleted. We previously performed a study on this phenomenon analyzing the experiments in PRIDE 29. In this study, we showed that some experiments available in PRIDE already contained a large portion of deleted identifiers at the time of their publication. If this effect is not taken into consideration, differences may appear that are only caused by changing protein accessions. Additionally, analysis pipelines generally use different protein inference algorithms as well as different models for protein homologues and isoforms 30 and potentially different false discovery rate thresholds 31—details that are often not reported. These differences in the data analysis will artificially introduce a high number of false‐positive differences between the compared samples. Therefore, a direct comparison of *final* proteomics results from different sources is possible but must be performed with extreme care.

This problem is even more pronounced in quantitative proteomics experiments. Labeled approaches only produce reliable quantification between the analyzed samples. It is not possible to directly compare reported intensities between different MS runs. In addition, label‐free quantification is highly dependent on the experimental protocol. The direct comparison of quantitative values based on label‐free approaches is in theory only possible if detailed information about the experimental procedures is available and similar across the compared experiments. In practice, even slight differences in chromatography and machine settings will prevent a reliable comparison of label‐free quantitative values without the use of a common reference. Therefore, the direct comparison of quantitative experiments on a large, unsupervised scale is currently not possible.

However, although challenging, we strongly believe that data reuse can accelerate clinical research considerably. The first author's previous research group was performing clinical proteomics studies at the Medical University of Vienna, which is linked to the General Hospital of Vienna, one of Europe's largest hospitals. The research group consisted of biochemists, analytical chemists, bioinformaticians, and clinicians working on rather diverse clinical questions. As a central point for the data analysis, we developed the Griss Proteomics Database Engine (GPDE) 32 as a database that merged single experiments based on the same disease and allowed the comparison of newly performed experiments with any number of previous ones. Thereby, we were able to use any or even all of our previously performed (unrelated) studies as controls for new studies. Recently, the GPDE led to the (unexpected) discovery that certain proteins indicating cisplatin resistance in melanoma cell lines were also found in certain multiple myeloma associated fibroblasts 33. In the initial analysis of melanoma cell lines, we identified 47 proteins that could indicate cisplatin resistance. To assess a biomarker panel's specificity, the GPDE has a function that quickly analyzes in which samples a panel of biomarkers was already detected. In this case, the tool showed that a subset of the 47 proteins from the panel was found in a total of six cell lines. We then performed subsequent experiments testing these cells sensibility to cisplatin. Surprisingly, we could show that the proportion of expressed proteins of the panel directly correlated with the cells’ cisplatin resistance. This finding not only provides strong evidence for the biomarker panel's predictive power and specificity, but also highlights an unexpected similarity between melanoma cell lines and multiple myeloma associated fibroblasts. This example demonstrates the possibility to increase our biological understanding by integrating heterogeneous datasets and thereby reduce the number of necessary experiments.

The GPDE was built around the PRIDE XML format, at that time the only vendor‐neutral format that could store MS data as well as identification data. It thereby inherently provides the possibility to seamlessly incorporate any data available in the PRIDE repository in an analysis. Nevertheless, this feature was never used. Most often, the lack of supplied metadata prohibited the selection of matching datasets. More importantly, objective, standardized measures of identification reliabilities were missing, prohibiting an automated import of data from PRIDE without the risk of considerably increasing the number of false‐positive identifications.

However, since then PRIDE has already taken several steps to explicitly support the reuse of submitted data. In this context, one of the most important resources is “PRIDE Cluster” 34. PRIDE Cluster uses a spectral clustering algorithm to create an independent assessment of reliable peptide and protein identifications. It clusters all identified spectra submitted to PRIDE to then identify reliable identifications. We could conclusively show that if at least 70% of the spectra within a (large enough) cluster were identified as the same peptide, that these can be considered reliable identifications 34. Thereby, the original submitted dataset is not reprocessed but left unaltered. Since PRIDE Cluster is using the comparison of spectra between independent experiments as a quality control method, the data quality and number of validated identifications rise with increasing data size. Thereby, additional experiments help to validate identifications made in previous ones. We are currently developing an updated version of the algorithm to manage the large increase in data through PX and be able to continuously integrate submitted experiments. Additionally, we are developing a new web interface to provide easier access to this information. This will enable researchers to quickly identify in which datasets a certain protein was identified—and through PRIDE Cluster whether this identification could already be validated. This resource may be a first step to enable researchers to integrate heterogeneous datasets into their analyses at the identification level.

PRIDE Cluster may gain even more importance in conjunction with the data‐independent acquisition approach SWATH‐MS 35. SWATH experiments can acquire the signals corresponding to theoretically all the peptides in a sample included in a range from around 400 to 1200 *m/z* units. Because several peptides are fragmented simultaneously, reliable identifications of peptides are only possible if their spectra were recorded before and are available in a spectral library. PRIDE Cluster automatically merges all reliable identifications from PRIDE and creates spectral libraries that have a comparable quality to the NIST's (National Institute of Standards and Technology) ones 34. Therefore, over time, these growing spectral libraries could potentially lead to additional identifications in SWATH experiments and thereby result in novel discoveries.

At this point, a study similar to the one by Yuan et al. 9 based on proteomics data seems not possible. The high heterogeneity in proteomics workflows makes the integration of proteomics *results* highly susceptible to artifacts produced by the different analysis workflows. The reanalysis of raw data from multiple sources is computationally challenging and can only be done by dedicated resources. The existing ones, such as PeptideAtlas, GPMDB, and ProteomicsDB, are not focused on the needs of clinicians and cannot be used to assist clinicians or clinical researchers to incorporate their results into their own analysis. For this task, dedicated resources are needed for specific diseases that can integrate the data from existing repositories to generate highly specific datasets. The standard, machine‐readable announcements of new PX datasets provide an ideal ground for the development of such resources. The Human Proteome Project has recently announced the launch of the “Biology/Disease‐driven Human Proteome Project” (B/D‐HPP) 36. We think this is an important step in the right direction and might provide such clinical specialized resources. However, sufficient metadata are the core prerequisite to a meaningful sample selection for a meta‐analysis as well as the creation of targeted resources. The currently possible way to select datasets of specific samples for performing meta‐analysis is through reading the associated published papers. This is possible for small, selected scenarios but not for any large‐scale data mining approach. As a first step, we have now introduced a new tag (“Biomedical dataset”) for clinically relevant PRIDE experiments. This new tag can be searched in PRIDE (http://www.ebi.ac.uk/pride.archive/simpleSearch?q = biomedical&submit = Search) and in ProteomeCentral. In addition, new PRIDE REST web services are being developed (available in beta at the moment of writing, http://www.ebi.ac.uk/pride/ws/archive/), enabling simple computational access to all data in PRIDE. Additionally, the EBI and the NCBI have created BioSamples focused databases 37, which can link datasets from the same sample analyzed using multiple techniques (i.e. proteomics data in PRIDE and genomics data in ArrayExpress). Thereby, particularly interesting datasets from a data reuse point of view are easier to access.

It is clear to us that we must focus our primary efforts on improving sample annotation in a meaningful way. Too high prerequisites will prevent researchers to submit their data, too little metadata will prevent a meaningful (re)use of the submitted data. We therefore need to find a balance that suffices both. In our opinion, this should be one of the main future focuses of PX. Such a refinement phase is a natural development, once the initial system has been set up and the initial requirements have been covered. A first vital step is the development of software tools that allow authors to easily annotate their processed result files—work that is now being planned at PRIDE for mzTab. Additionally, reviewers must be provided with methods to verify that the required annotation is present and correct. At last, journals in collaboration with repositories are essential to enforce a minimal amount of metadata in the deposited datasets. Otherwise, invaluable experimental data may be lost to elicit future findings.

## Supporting information

As a service to our authors and readers, this journal provides supporting information supplied by the authors. Such materials are peer reviewed and may be re‐organized for online delivery, but are not copy‐edited or typeset. Technical support issues arising from supporting information (other than missing files) should be addressed to the authors.

Table
